# Learning from clinicians’ views of good quality practice in mental healthcare services in the context of suicide prevention: a qualitative study

**DOI:** 10.1186/s12888-019-2336-8

**Published:** 2019-11-06

**Authors:** Donna L. Littlewood, Leah Quinlivan, Jane Graney, Louis Appleby, Pauline Turnbull, Roger T. Webb, Navneet Kapur

**Affiliations:** 1 NIHR Greater Manchester Patient Safety Translational Research Centre, The University of Manchester, Manchester, UK; 2 Manchester Academic Health Science Centre (MAHSC), The University of Manchester, Manchester, UK; 3 National Confidential Inquiry into Suicide and Safety in Mental Health (NCISH), Centre for Mental Health and Safety, School of Health Sciences, The University of Manchester, Manchester, UK; 4 Greater Manchester Mental Health NHS Foundation Trust, Manchester, UK

**Keywords:** Mental health services, Suicidal behaviour, Suicide prevention, Qualitative research, Patient safety, Quality

## Abstract

**Background:**

Suicide and non-fatal self-harm represent key patient safety events in mental healthcare services. However, additional important learning can also be derived by highlighting examples of optimal practice that help to keep patients safe. In this study, we aimed to explore clinicians’ views of what constitutes good practice in mental healthcare services in the context of suicide prevention.

**Methods:**

Data were extracted from the National Confidential Inquiry into Suicide and Safety in Mental Health (NCISH) database, a consecutive case series study of suicide by people in contact with mental healthcare services. A large national sample of clinicians’ responses was analysed with a hybrid thematic analysis.

**Results:**

Responses (*n* = 2331) were submitted by clinicians across 62 mental healthcare providers. The following five themes illustrated good practice that helps to: 1) promote safer environments, 2) develop stronger relationships with patients and families, 3) provide timely access to tailored and appropriate care, 4) facilitate seamless transitions, and 5) establish a sufficiently skilled, resourced and supported staff team.

**Conclusion:**

This study highlighted clinicians’ views on key elements of good practice in mental health services. Respondents included practice specific to mental health services that focus on enhancing patient safety via prevention of self-harm and suicide. Clinicians possess important understanding of optimal practice but there are few opportunities to share such insight on a broader scale. A further challenge is to implement optimal practice into routine, daily care to improve patient safety and reduce suicide risk.

## Background

Suicide is a key patient safety concern that accounted for 17,931 deaths of UK mental healthcare patients between 2006 and 2016 [1]. Improvement in the quality and safety of mental healthcare services is a fundamental aspect of suicide prevention. In England, action to reduce suicide risk among mental health patients features saliently in the National Suicide Prevention Strategy [2]. Much of the work in this area is driven by post-suicide investigations that seek to review and glean insight from the antecedent clinical practice by identifying instances where care could be improved [1, 3].

In the UK, this work is conducted systematically by the National Confidential Inquiry into Suicide and Safety in Mental Health (NCISH), which collects detailed data pertaining to people who died by suicide and who accessed mental healthcare services during the preceding 12 month period. To date, NCISH has identified suboptimal clinical care and other relevant antecedents, and have consequently made practice recommendations aimed at preventing future suicide. Indeed, NCISH recommendations such as the removal of ligature points in inpatient settings, and implementation of 7 day follow-up on discharge from an inpatient unit, have been linked with a subsequent reduction in patient suicide rates [1, 4, 5]. Patient safety research has traditionally taken a reactive approach, focused on reviewing care preceding a serious adverse patient outcome to identify modifiable suboptimal practice. However, to further our understanding of safe and effective practice it is also important to proactively identify everyday care associated with positive patient outcomes [6, 7].

For instance, people who accessed psychiatric in-patient care in relation to suicidal experiences have highlighted key aspects of care perceived to contribute to recovery [8]. Participants emphasised the importance of trusting, therapeutic relationships with clinicians, who see them as a valuable person and who provide tailored, individualised care [8]. In addition to patient insights, clinicians possess a wealth of experiential knowledge which has proved valuable to the improvement of the quality and safety of services [9]. A recent qualitative investigation of 18 heads of emergency and psychiatry departments identified practice that was perceived to facilitate improved access to psychiatric consultation and referrals for people who have attempted suicide [10]. Consequently, the NCISH questionnaire adopts a comprehensive approach to investigating safe mental health care, seeking to identify practice that may have prevented patient deaths by suicide [1], and also highlight aspects of broader everyday care that constitutes safe and effective practice. This novel and nationally representative dataset has provided a key opportunity to learn from examples of good practice identified in reviews of care provided by mental healthcare services.

### Aims

The study reported herein aimed to examine staff views of good practice in mental healthcare services in England using nationwide data collected by NCISH. It thereby provided a unique opportunity to derive insight from the accumulated clinical wisdom of mental healthcare staff accrued through extensive practical experience.

## Method

### Design

NCISH is a UK-wide consecutive case series study of all deaths by suicide among people in contact with mental healthcare services (including inpatient units, crisis and home treatment teams, community mental health teams, psychological services). In our study, we analysed qualitative data collected via the NCISH questionnaire, which relate specifically to clinician views of good practice within mental healthcare services. Ethical approval was granted for the NCISH project by the South Manchester Medical Research Ethics Committee, the North West Research Ethics Committee, the National Information Governance Board for Health and Social Care, the Patient Information Advisory Group, and approval under Section 251 of the Mental Health and Social Care Act.

### Case ascertainment

Data collection has been extensively described previously [11]. In brief, NCISH collect data for all individuals in the United Kingdom aged 10 years and older who: 1) have died by suicide, and 2) were in contact with specialist mental healthcare services during the preceding 12 month period. A questionnaire is used to capture sociodemographic information and clinical antecedents to a patient’s death by suicide. The questionnaire is sent to the consultant psychiatrist who had been responsible for the patient’s care, and is typically completed by a senior clinician(s) who was part of the patients care team who may also extract information from the patients care records [12]. In 2011, NCISH added an additional item to solicit examples of good quality practice in mental healthcare.

### Data analysis

Data collected in response to the NCISH questionnaire item on good practice in mental healthcare services (*Can you give examples of good practice in your service that others might adopt?*) were analysed. Data were available for questionnaire returns made in relation to patient’s death by suicide that occurred from 1st January 2011 to 31st December 2016. To minimise heterogeneity in service delivery models and policy, the data were restricted to responses for patients who, 1) died in England, 2) were aged 18 years or over, and 3) were not in prison when they died.

Data were analysed by a multidisciplinary team with research and clinical expertise in mental health, health services research, and qualitative methodologies. Thematic analysis was conducted using a hybrid approach, [13] whereby the data were coded deductively, with a pre-determined coding framework developed from clinical guidance for practice in mental healthcare services, and inductively to ensure additional pertinent codes were included within the analysis. The deductive coding framework was developed from relevant patient safety focussed clinical guidance, namely, the NCISH recommendations for ‘10 Key Elements To Improve Safety’ [14, 15] and the National Institute for Health and Care Excellence (NICE) Self-harm Quality Standard - QS34 (provided in Additional file 1). These codes were then reviewed and consolidated, where appropriate, to provide the initial, deductive coding framework.

The first and second authors (DLL and LQ) of this manuscript coded the dataset independently using the deductive framework, and developed new inductive codes as necessary. Throughout the coding process these two researchers met periodically to review and revise both the newly developed inductive codes and the application of the deductive coding framework. Preliminary themes were developed by grouping codes according to their similarities and differences. Discrepancies were resolved by returning to the dataset to test and refine theme descriptions. An overview of the preliminary thematic structure, including descriptions of each theme, was provided to the third author (JG). She coded a 10% (*n* = 233) sample of the data at a thematic level to assess the extent to which the thematic structure could be applied consistently to reflect the patterns across the dataset [16]. This sample was generated using a random-stratified approach to ensure even representation from across each of the themes. Two of the authors (DLL and JG) then reviewed convergence between coding, which led to the revision and expansion of the theme descriptions to enhance the clarity of the boundaries between themes. The final thematic structure was agreed through group discussion between authors (DLL, LQ, JG, RTW, NK).

## Results

During the sampling period, questionnaires were completed in relation to 7066 patient deaths by suicide. Of which, 2331 questionnaires provided data regarding their views of good practice. Responses were submitted by 62 mental health providers (57/62 = National Health Service [NHS] mental health service providers, 5/62 = independent providers). These providers submitted a median of 33 responses (IQR = 30^.^5) during the sampling period, with the number of responses provided per provider ranging from 1 to 98. Ninety-eight per cent of all responses came from NHS mental health service providers (*n* = 2286). Responses were provided from service providers from all geographical regions across England. Twenty-three per cent of respondents specified their occupation (*n* = 527/2331), of whom 44% were consultant psychiatrists, 25% were in service management positions, 12% were mental health practitioners (including nurses, social workers, occupational therapists), 9% were doctors, 7% were psychologists, and 3% reported a wide range of other occupations. Responders provided a wide range of examples of good practice, with some extracted from clinical notes that detailed the historical care provided to patients who had died by suicide, e.g. “*client had open access to telephone care coordinator*,” and others stemming from their broader clinical experience, e.g. “*printed leave care plan with details of relapse signature, ward contact details, etc. given to all patients and their carers who go on leave*.” Five themes captured the aspects of mental healthcare that were described as evidencing good practice. Descriptions of the themes with illustrative data extracts are provided in Table 1. Study assigned provider ID numbers are included as descriptors alongside the extracts.

**Table 1:** Aspects of mental healthcare services that staff perceived as good practice

Theme	Description of theme.	Exemplar clinician responses
Promote safer environments	This theme centred around practice on inpatient wards and safely managing leave or discharge from the wards. Examples of good practice included: • Good quality observations conducted by trained staff. • Assessment of risk and mental state conducted prior to leave or discharge. • Collaboratively developing safety, crisis and contingency, and leave plans, which includes access to support from crisis and home treatment teams. • Copies of leave plans provided to all parties involved in the patient’s care (incl. Patients and families), and include contact details for services. • Follow up contact with patients during leave from wards. Effectively managing access to medication was seen as playing an important role in promoting safer environments. Safer prescribing practices included reduction in the supply of medication provided to patients to reduce risk of overdose. The care team should also communicate with patients’ GPs to plan and coordinate access to medication. Finally, ‘no blame’ open learning cultures that provide the opportunity to review practice following patient deaths by suicide were also identified as constituting good practice.	*“Observation training for it to be engaging and therapeutic”* (ID43) *“Risk assessment carried out for every period of leave”*(ID14) *“Use of collaboratively created crisis plans to support out of hours care”* (ID57) *“Printed leave care plan with details of relapse signature, ward contact details etc given to all patients and their carers who go on leave”* (ID21) *“Daily telephone contact with patients who are on overnight leave”* (ID61) *“Very close liaison with the GP to prevent obtaining double prescriptions”* (ID59) *“We include regular ‘learning lessons’ feedback where care can be improved and where care has gone well in our clinical improvement and business meetings”*(ID61) *“We have an open culture to discuss and reflect from SI [serious incident] and new events”* (ID42)
Develop strong relationships with patients and family/carers	Developing strong relationships with patients and their families and carers was seen as a vital part of delivering good quality mental healthcare services. Good practices emphasised by respondents included: • Active involvement of patients and their partners, families and carers in both care planning and the provision of care. • Seek to build rapport with patients and maintain regular contact, as appropriate for the patient’s current level of need. • Provide continuity of care by establishing consistency in the healthcare team, often through the assignment of a keyworker (e.g. care-coordinator). • Adopt a more proactive approach to engaging patients with services, particularly in the case of proactively following up patients who miss appointments. • Dedicated outreach service focussed on providing intensive support to enhance patients’ levels of engagement with services. • Develop strong relationships with families that include two-way communication and information sharing. • Responsive to family members’ concerns and staff share concerns with family members when patients missed appointments, or were not complying with medication. • Provide support to family members in relation to their own health needs, as part of a family intervention including the patient, or in the event of a patient’s death by suicide.	*“Active involvement of patient’s family in discharge planning”* (ID15) *“His care co-ordinator knew him very well, had regular contact, there were clear efforts to try and have frequent contact with him”* (ID55) *“Same consultant for inpatient and CRHT [Crisis Resolution & Home Treatment team] maintained continuity of care and communication”* (ID57) *“Assertive outreach remaining I think the gold standard for providing intensive, multi-disciplinary treatment with continuity”* (ID6)” *“Although discharged from HTT [home treatment team] the team did unplanned visit when his relative reported him missing”* (ID47) *“Support for the family after patient’s death”* (ID55)
Provide timely access to tailored and appropriate care	This theme centred around providing timely access to tailored support and treatment including: • Prompt access to assessments, appropriate support, and treatment. • Tailored needs-based care with active patient involvement in developing person-centred care plans and decision making in relation to their care. • The adoption of a holistic multi-agency approach to care that also considers the patient’s physical health and psychosocial needs. Clinicians also championed the provision of evidence-based specialist support and treatment that are aligned with national policies and guidelines, including access to: • Psychological services and therapies such as Acceptance and Commitment Therapy, Cognitive Behavioural Therapy, and Dialectical Behaviour Therapy; • Specialist alcohol and substance misuse services • 24/7 crisis resolution and home treatment teams and crisis houses.^a^ Detailed and routinely updated assessments were also perceived as good practice, including the assessment of safety, risk, and mental capacity. The ability for patients to easily re-access mental healthcare services, without having to endure long referral times, was also important.	*“Easy/quick access to treatment”*(ID11) *“Patient’s wishes were taken into consideration”* (ID60) *“Good liaison with housing department, input from employment specialist”* (ID59) *“Provide a high quality, evidence-based service in line with national and local policies and guidelines”* (ID60) *“Availability of psychology service in both primary and secondary care setting”* (ID24) *“Crisis service is 24 h 7 days a week service, response to emergency referrals is usually within 2 h”*(ID52) *“Repeated mental state examinations and risk assessments”* (ID54) *“[.] discharged patients [have] rapid access back into services when they relapse;* i.e. *no need for GP referral triage or allocation”* (ID35)
Facilitates seamless transitions	This theme highlighted the importance of effective communication practice that facilitates seamless transitions between, and discharge from services. Practices included: • Care planning should include and be communicated with the relevant care team and other health and social care providers, particularly the patient’s GP. • Patient notes should be up-to-date and accessible to all staff teams involved in providing care. • Follow up contact with patients post transition/ discharge. • Consistency of staff across transition, e.g., staff from new service introduced prior to transition to their service.	*“Discharge/transfer of care plans to be communicated with GP and the relevant services”* (ID41) *“Electronic records across all treatment services in the trust which allows immediate access”* (ID51) *“Clear discharge planning on leaving inpatient unit, with onward referrals and follow ups made”* (ID32) “*Implementation of key worker system within Crisis Teams which designates a specific worker to oversee the patient’s care and transition to ongoing service*” (ID50)
Establish a sufficiently skilled, resourced and supported staff team	Having sufficient staff with the appropriate mix of skills and expertise was perceived as an essential aspect of delivering good practice. Respondents saw value in: • Staff having time and capacity to build relationships and cover absence in order to meet patient needs. • Multi-disciplinary teams within service, plus provision of input from wide range of expertise and specialist disciplines, such as psychologists, and occupational therapists. • Regular and timely access to input from consultant psychiatrists. • Staff expertise in assessment and formulation. Clinicians also highlighted the importance of addressing staff needs through: • Meeting the training, development and support needs of staff. • Providing regular clinical supervision including observation of practice, and having the opportunity for debriefing and reflective practice. • Offering support following a patient’s death by suicide	*“Urgent cover offered when care coordinator not available to meet needs of patient”* (ID13) *“Multidisciplinary team approach including psychology, recovery, wellbeing and care coordination”* (ID28*)* *“Was seen by the consultant within hours after initial assessment”* (ID29) *“Regular supervision and at agreed intervals”* (ID51) *“Increased emphasis on training and education in suicide prevention”* (ID25) *“Staff support following suicide”* (ID46)

^a^In the UK, the specific set up of Crisis houses vary, but they generally provide intensive, short-term support to people during a mental health crisis. Typically, they offer support in a home-like setting and act as an alternative to psychiatric hospital care. Crisis houses are only available in some areas of the UK and may be provided either by the NHS or by an independent provider

## Discussion

This study has provided novel insight into perceptions of good mental healthcare practice from the perspective of a large, nationwide sample of clinicians, commenting on optimal care after patients have died by suicide. With the exception of the ‘promoting safer environments’ theme, the areas highlighted by clinicians constituted quality practice irrespective of the healthcare setting. However, context is an important distinguishing feature, in that failure to deliver safe, optimal care can have seriously harmful consequences for patients in mental healthcare services. For instance, failure to ‘develop strong relationships with carers’ and to ‘provide timely access to appropriate care’ are associated with catastrophic patient safety outcomes, such as suicide [1, 3–5, 14, 17].

### Good practice specific to mental healthcare services

One theme described good practice that is specific to mental healthcare settings, namely, ‘promote safer environments’. Respondents emphasised a range of practices that aim to improve safety both in a ward environment, and in the community via the use of safety and crisis plans, and managing access to medication. Practice outlined by clinicians in this theme is consistent with NCISH recommendations for ‘10 Key Elements To Improve Safety’ which stem from data collected in relation to over 33,500 patients who have died by suicide [14]. It is possible that this consistency stems from the dissemination of NCISH recommendations having influenced clinicians understanding of good practice. To date there has been no systematic evaluation of the extent to which NCISH recommendations are routinely adopted into everyday practice. However, implementation of these recommendations has been linked to improved patient safety, as evidenced by a subsequent reduction in suicide risk [4, 5, 14]. Future research should solicit clinician and patient views on the specific NCISH recommendations they perceive to be most vital to preventing suicide and improving patient wellbeing.

### Patient safety and the importance of good practice in mental healthcare services

Good practice outlined in the remaining themes broadly applies to all healthcare settings, e.g., ‘developing strong relationships with patients and carers’, and ‘providing timely access to tailored and appropriate care*’*. However, there are important contextual differences in providing safe, high quality care in mental healthcare services, where the majority of patient safety incidents relate to self-harm, aggressive behaviours, restraint, absconding and reduced capacity for self-advocacy [18, 19]. Thus, the consequences of failing to provide good practice in mental healthcare service settings can have serious implications for patients. This can be illustrated by considering the consequences of failings to 1) effectively involve patient’s families and carers, and 2) provide timely access during a mental health crisis.

Family involvement is of particular importance to patient safety in mental healthcare service settings, where communication with and involvement of family members may help to prevent patient deaths by suicide [1, 3, 17]. In our study clinicians provided specific examples of family involvement practice that were perceived to have enhanced patient safety. The value of two-way dialogue was emphasised, whereby family members feel able to share their concerns about a patient’s current wellbeing and safety, which consequently leads to action by the healthcare team. Equally, clinicians involved family members by informing them when patients missed appointments, or were not complying with medication. However, family involvement can be challenging in mental healthcare settings [20–22]. Whilst families can provide an effective source of support to some patients, for others, difficult family relationships may contribute to their distress. Furthermore, staff report difficulties in negotiating confidentiality and privacy, [20] which can hinder information sharing [3]. Future research should seek to provide a nuanced understanding of how these barriers to involving families can be effectively overcome so that involvement is adopted into routine, everyday care.

The implementation of ‘timely access to tailored and appropriate care’ was emphasised by respondents, which included the timely provision of evidence-based specialist support such as psychological therapies, and crisis resolution and home treatment. In accordance with this, access to 24-h crisis services has been linked to reduced suicide risk [5]. However, the implementation of waiting-time targets and access standards for mental healthcare services in England has lagged behind those established for physical healthcare, such as cancer treatment, or surgical interventions [23]. Recent progress has been made through the establishment of access standards for Improving Access to Psychological Therapy (IAPT), early intervention in psychosis, and children and young people’s eating disorder services [24]. Plans are currently in development to introduce similar waiting-time access standards for adult community mental health teams and emergency mental healthcare services [24].

### Strengths and limitations

We analysed a large nationally representative dataset of 2331 clinicians’ responses collected via the NCISH questionnaire. The analysis was conducted by a multidisciplinary team that included both clinical (nursing and psychiatry) and qualitative research expertise. Analysis that is conducted by a research team encompassing diverse expertise and viewpoints is commonly referred to as ‘researcher triangulation,’ which serves to enhance the robustness and validity of the analysis [25]. The NHS Resolution report, ‘Learning from suicide-related claims’ indicated that findings from serious investigations post-suicide are not effectively shared with other Trusts at a national level [3]. Our study utilised the NCISH database to facilitate knowledge sharing across the NHS, by reporting unique insight drawn from a large, nationwide sample of clinicians from NHS mental healthcare service trusts throughout England.

The reported findings should, however, be considered in the context of three main limitations. First, the study data pertained only to clinicians who provided their views as part of a broader questionnaire following the suicide of a mental health patient. However, the data were drawn from responses to a more generic question about perceptions of good practice in mental healthcare services. Consequently, the extent to which responses represent views of good quality practice that contributes to suicide prevention versus quality care in general, remains unclear. That said, patient safety is defined as one of the three core areas of quality healthcare, alongside clinical effectiveness and patient experience [26]. It is likely that the themes identified here may contribute to improved patient safety via a broader approach to enhanced quality of care. Second, it is important to acknowledge that the practice outlined is based on clinician perceptions solicited as part of a wider investigation following a patient’s death by suicide. It is possible that this context may bias responses. Third, due to the data collection method, it was not possible to clarify or seek expansion on any ambiguity in clinicians’ responses, or to examine the specific practice perceived to be most salient to providing quality care. It was also beyond the scope of the current study to assess the extent to which the practices reported are associated with improved patient outcomes. Having obtained a large sample of clinician views about good practice, conducting focus groups with clinicians may prove a useful next step to identify key practices that can be implemented across services, irrespective of constrained resources. In addition, sampling approaches should be expanded in future research to include the views of patients and their families and carers.

### Clinical implications

Though we cannot assert that practices outlined by clinicians accurately reflect effective practice, it is somewhat encouraging that views revealed in this study concur with evidence-based recommendations from NCISH, and NICE clinical guidance. Therefore, it is important to consider what factors act as barriers to the implementation of such practice into routine care. Respondents described a wide-range of practice that extends beyond those tied directly to availability of resources. Nonetheless, insufficient resources might be one impediment to the implementation of quality practice. For instance, implementing the timely access standards for mental healthcare services may prove challenging due to insufficient staffing and other resources. In a recent survey of finance directors at NHS mental healthcare trusts, 80% reported that financial pressures had contributed to longer waiting times for accessing services, and inadequate incapacity to offer a full range of recommended treatment options [27].

A rising staff turnover rate is a major concern that not only negatively affects access to care, but also hinders the delivery of quality care more broadly. Staff turnover rates have risen across mental healthcare in the NHS, which equates to a loss of over 10,000 mental health staff each year [28]. Moreover, previous research conducted by our team reported an association between staff turnover and patient suicides rates [4]. In addition, the implementation of safety improvement recommendations was associated with greater reductions in suicide rates at providers with lower levels of staff turnover [4]. Healthcare systems are complex, and whilst access to sufficient resources undoubtedly has key implications for the quality of healthcare provision, it is not the sole factor that influences the adoption of evidence-based clinical guidance into routine care. Furthermore, this study highlights the depth of experiential knowledge developed by clinicians and the value in providing opportunities for them to share their insight to benefit colleagues and practice on a wider scale. Therefore, translational research should seek to learn from instances where good practice has been effectively implemented into everyday care and provide mechanisms for clinicians to share insight more broadly.

## Conclusion

Previous insight into effective healthcare practice has been derived by focusing solely on identification of suboptimal care through post-incident reviews. This study adopts an alternative approach and presents the first nationwide investigation of clinicians’ views of optimal practice in mental healthcare services. Our findings highlight care perceived to be effective in improving patient outcomes. Specific practices were described by clinicians that seek to reduce suicide risk, such as taking action to promote safer clinical and community environments for patients. This study also illustrates the value in providing opportunities for clinicians to share their experience in order to support quality improvement within mental healthcare.

## Supplementary information

**Additional file 1.** Deductive code descriptions derived from NICE Self-harm Quality Standard (QS34) and NCISH 10 Key Elements to Improve Safety. List of all codes deducted from the aforementioned NICE and NCISH recommendations.

## Data Availability

The National Confidential Inquiry case series database is not publically available. For details further details about the project and dataset please visit: https://sites.manchester.ac.uk/ncish/

## Abbreviations

NCISH: National Confidential Inquiry into Suicide and Safety in Mental Health
NHS: National Health Service
NICE: National Institute for Health and Care Excellence
