# Supplementary material for: Learning from clinicians’ views of good quality practice in mental healthcare services in the context of suicide prevention: a qualitative study
Source: BMC Psychiatry. 2019 Nov 6;19:346. doi: 10.1186/s12888-019-2336-8 (PMC6836656; doi:10.1186/s12888-019-2336-8)
Supplement: Supplementary file 1 — Additional file 1. Deductive code descriptions derived from NICE Self-harm Quality Standard (QS34) and NCISH 10 Key Elements to Improve Safety. List of all codes deducted from the aforementioned NICE and NCISH recommendations. [file 12888_2019_2336_MOESM1_ESM.docx]

Additional file 1

**Deductive code descriptions derived from NICE Self-harm Quality Standard (QS34) and NCISH 10 Key Elements to Improve Safety**

| **Codes derived from NICE Self-harm Quality Standard - QS34** |
| --- |
| Staff training includes treating people with compassion, respect and dignity that includes specific reference to people who self‑harm. |
| People who have self‑harmed are cared for with compassion and the same respect and dignity as any service user |
| Initial assessments of people who have self‑harmed include physical health, mental state, safeguarding concerns, social circumstances and risks of repetition or suicide. |
| People who have self‑harmed receive a comprehensive psychosocial assessment |
| People who have self‑harmed receive the monitoring they need while in the healthcare setting, in order to reduce the risk of further self‑harm |
| Undertake environmental assessments of healthcare settings, including assessing the risks of environment to people who have self‑harmed. |
| Collaboratively developed risk management plan for people receiving continuing support for self‑harm |
| Access to psychological interventions specifically structured for people who self‑harm |
| People receiving continuing support for self‑harm and moving between mental health services have a collaboratively developed plan describing how support will be provided during the transition |
| **Codes derived from NCISH 10 Key Elements to Improve Safety** |
| Removal of ligature points |
| Measures in place to prevent leave from ward without staff agreement |
| In-patient observation is a skilled intervention to be carried out by experienced staff |
| Early follow up on discharge from hosptial to community within 2 - 3 days |
| Care plan in place at the time of discharge from acute care |
| No 'out-of-area' admissions |
| Provision of 24 hour crisis resolution/ home treatment teams |
| Community outreach service available |
| Specialised alcohol and drug misuse service available |
| Multidisciplinary reviews following all suicide deaths to include input and information sharing with families/carers |
| Services should work closely with families throughout care pathway |
| Policy based on NICE guidance on depression |
| Policy based on NICE guidance on self-harm |
| Personalised risk management |
| Low turnover of non-medical staff |
